# Influence of cytochrome P450 2D6*10/*10 genotype on the risk for tramadol associated adverse effects: a retrospective cohort study

**DOI:** 10.3389/fphar.2024.1358549

**Published:** 2024-02-19

**Authors:** Mahmood Mahajna, Rami Abu Fanne, Mahmoud Odeh, Matitiahu Berkovitch, Elias Tannous, Sara Eyal, Shlomo Vinker, Ilan Green, Ilan Matok

**Affiliations:** ^1^ Department of Clinical Pharmacy, The Hebrew University, Jerusalem, Israel; ^2^ Hillel Yaffe Medical Center, Hadera, Israel; ^3^ Department of Cardiology, Hillel Yaffe Medical Center, Hadera, Israel; ^4^ Leumit Healthcare Services, Tel Aviv, Israel; ^5^ Department of Clinical Pharmacology and Toxicology, Shamir Medical Center Affiliated with Sackler Faculty of Medicine, Tel Aviv University, Tel Aviv, Israel; ^6^ Department of Medical Sciences, Faculty of Medicine, Ben-Gurion University of the Negev, Beersheva, Israel; ^7^ Institute for Drug Research, Faculty of Medicine, The Hebrew University, Jerusalem, Israel

**Keywords:** CYP2D6, database, O-desmethyltramadol, polymorphism, tramadol

## Abstract

**Background:** Tramadol is primarily metabolized by the highly polymorphic *CYP2D6* enzyme, leading to a large spectrum of adverse events and clinical response. Ample evidence pointed a reduced CYPD26 activity score in individuals harboring the CYP2D6*10/*10 genotype, nevertheless, there is scarce studies on the impact of CYP2D6*10/*10 genetic polymorphism on long-term tramadol’s adverse effects.

**Aim:** To test the correlation between CYP2D6*10/*10 expression and the risk for tramadol-associated adverse effects.

**Method:** Using a database of Leumit Healthcare Services in Israel, we retrospectively assessed the occurrence of adverse events in patients who were prescribed tramadol. A binary logistic regression model was applied to model the relationship between CYP2D6*10/*10 genotype and the occurrence of adverse effects.

**Results:** Data from four hundred ninety-three patients were included in this study. Only 25 (5.1%) patients were heterozygous for the CYP2D6*10 variant, while 56 patients (11%) were tested positive to the CYP2D6*10/*10 genotype. Compared to carriers of other variants, patients with the CYP2D6*10/*10 variant exhibited a higher occurrence of adverse events (odds ratio [OR] = 6.14, 95% confidence interval 3.18–11.83); the odds ratio for central nervous system adverse events and gastrointestinal adverse events were 5.13 (95% CI 2.84–9.28), and 3.25 (95% CI 1.78–5.93), respectively.

**Conclusion:** Among the different *CYP2D6* genotypes, CYP2D6*10/*10 genotype carries the higher risk of tramadol related adverse events. Appreciating the frequency of this specific allele it seems prudent to pharmacogenetically screen patients considered for long term tramadol treatment for better tolerability and efficacy outcomes.

## Introduction

Tramadol is a centrally-acting opioid analgesic that is widely used in the treatment of acute and chronic pain and is the most commonly used opioid worldwide (Mohammadpour et al., 2019). Despite being an effective painkiller, tramadol can induce numerous side effects including respiratory depression, coma, seizures, vomiting, nausea, dizziness, drowsiness, headache, sedated state, abdominal discomfort, decreased appetite, and serotonin syndrome (Stassinos et al., 2017; Faria et al., 2018).

Tramadol exists as a racemic mixture consisting of two pharmacologically active enantiomers that both contribute to its analgesic effect through different mechanisms (Saarikoski et al., 2015). Tramadol is metabolized into active and inactive metabolites and is eliminated mainly in the urine, 30% as the unchanged drug and 60% as metabolites (Wu et al., 2002).

Genetic polymorphism has emerged as a key player in explaining phenotypic differences amongst individuals, including disease risk and drug response. This was elegantly proved in the case of tobacco consumption and effects, as a major public health issue. Nicotine is primarily metabolized by the CYP2A6 gene products. Genotypic polymorphisms of CYP2A6 have a significant impact on smoking behavior–individuals with low CYP2A6 activity score consume fewer cigarettes per day and over their lifetime and have a lower risk of lung cancer. CYP2A6 gene expression was considered as a possible genetic marker for the prevention and treatment of nicotine addiction, including Cancer susceptibility (López-Flores et al., 2017). In the case of tramadol, it is extensively metabolized by CYP2D6 with considerable interindividual variability. Cytochrome P-450 (CYP2D6) mediate the metabolism of tramadol to the primary active metabolite (M1) O-desmethyltramadol (ODT), while N-desmethyltramadol (M2) is catalyzed by CYP2B6 and CYP3A4. M1 and M2 may then be further metabolized to secondary metabolites, N, N-didesmethytramadol (M3), and N, O-didesmethyltramadol (M5) (Ryan and Isbister, 2015). The affinity of ODT to the mu µ) opioid receptor is 300-fold higher than that of its parent compound (Mohammadpour et al., 2019). These mechanisms are complementary and synergistic, improving tramadol’s ability to control pain relief (Stassinos et al., 2017).

The CYP2D6 polymorphisms has a major impact on the pharmacokinetics and pharmacodynamics of tramadol. Previous studies have shown that carriers of the CYP2D6*10 genotype, have reduced CYP2D6 activity and exhibit lower plasma concentration of the tramadol active metabolite (ODT) than normal metabolizer and cause fewer adverse effects (Crews et al., 2021). Likewise, the median plasma area under the curve (AUC) value for ODT was 0 (range 0–11) ng h/mL and 67 (range 17–118) ng h/mL, in poor and normal metabolizers, respectively (Stamer et al., 2007). Similarly, in healthy volunteers receiving a single dose of tramadol, ultrarapid metabolizers had a 7% higher AUC for ODT than normal metabolizers, and greater analgesia and adverse effects (Kirchheiner et al., 2008). In contrary, a recent population pharmacokinetic study of tramadol and its metabolites in healthy Korean subjects showed that the peak plasma concentration of tramadol in CYP2D6*10/*10 carriers is higher than normal metabolizers, and they speculated this loss of function genotype might be associated with higher incidence of tramadol related adverse effects compared to carriers of the frequent alleles (Lee et al., 2019).

The CYP2D6*10 variant is frequent in East Asians (45%), South Central Asians (20%), and Middle East individuals (5.5%) (Gaedigk et al., 2017). In the Israeli population, the major variants are *1, *2 (normal function), *3, *4, *5 (no function), *10, *17, and *41 (decreased function) (Bradford, 2002; Gaedigk et al., 2017; Koopmans et al., 2021). Among the studied Israeli populations, the highest frequency of CYP2D6*10 allele was found in Yemenite Jews (16.6%); this rate is lower than that of Asians but higher than that of Sephardic Jews (6.4%), Ethiopian Jews (5.4%), and Bedouins (2.0%) (Luo et al., 2004). Notably, individuals who are homozygous for CYP2D6*10/*10 are defined as intermediate metabolizers (IMs) (Caudle et al., 2020).

Although reporting higher tramadol concentration among CYP2D6*10/*10 variants, the Korean population pharmacokinetic analysis study, did not address the occurrence of adverse effects based on the inferred metabolizing phenotype. The current study was designed to assess for possible correlation between the CYP2D6*10 variant and the risk for tramadol-associated adverse effects.

## Methods

### Study design

This was an observational, retrospective, longitudinal cohort study. It included patients registered in the Leumit Healthcare Services (LHS) database, one of four Israeli Health Maintenance Organizations. The study was conducted in accordance with the declaration of Helsinki, and the protocol has been reviewed and approved by the Helsinki Committee of Leumit Healthcare Services (reference: 0225–18). Due to the retrospective nature of this study, the need for informed consent was waived.

### Setting and population

LHS registered outpatients, aged 21–90 years who started treatment with oral tramadol for acute or chronic pain were allocated during the study period. All patients included had an available blood *CYP2D6* genotyping result. The duration of the study was 6 years, from 1 January 2013, to 31 December 2018. Only patients with accurate documentation of the drug administration including drug dose at baseline were included in this study. Patients reached the study endpoint if they were transferred to another healthcare service, died, or remained at the end of the study data collection period.

### Genotyping

Blood samples for genetic tests were previously measured as part of a project to improve the quality of care and promote a personalized medicine approach in Leumit Healthcare Services (protocol number 144/11). Briefly, patients’ identification and their blood samples were coded to an identification code. The samples were collected by Teva Pharmaceutical Industries Ltd., which delivered the samples to the Genelex Laboratory in the United States of America. Genelex, is accredited by the College of American Pathologists (CAP 1073709) and licensed to perform high-complexity clinical testing in all U.S. states. Genotypes were obtained using a laboratory developed multiplex polymerase chain reaction–based test followed by a single base primer extension assay for variant detection by the MassARRAY system (Agnea Biosciences, San Diego, CA). This test identifies 17 small nucleotide variants and two gene rearrangements in PCR-multiplex format, providing increased sensitivity and quality performance. This CYP2D6 Mutation Detection Panel included PCR based assays to detect all common and rare variants with known clinical significance at analytical sensitivity and specificity greater than 99% (Azhar Gohar et al., 2016). Analytical specificity and sensitivity for detection of these mutations are >99%. This CYP2D6 Mutation DNA Analysis test has a Current Procedural Terminology (CPT) code of 81,226. The tested CYP2D6 (cytochrome P450, family 2, subfamily D, polypeptide 6) alleles included among others: *1, *2, *3, *4, *5, *6, *9, *10, *17, *19, *29, *35, *41, *1XN, *2XN, *4XN. The results of the genetic tests were transmitted electronically to the study coordinator, who maintained data accuracy using the pre-assigned code. Then, all samples were sent back to Shamir Medical Center for secure storage under appropriate conditions according to the Israeli regulations. The CYP2D6 activity score was expressed by a standardized phenotype classification system: CYP2D6 normal metabolizer (e.g., CYP2D6*1/*10, *1/*41, *1/*9, *10/*41x3, *1/*1, *1/*2, *2x2/*10) has activity score range (1.25–2.25), CYP2D6 intermediate metabolizer (e.g., CYP2D6*4/*10, *4/*41, *10/*10, *10/*41, *41/*41, *1/*5) has activity score range (0–1.25), CYP2D6 poor metabolizer (e.g., CYP2D6*3/*4, *4/*4, *5/*5, *5/*6) has activity score 0), and CYP2D6 ultrarapid metabolizer (e.g., CYP2D6*1/*1xN, *1/*2xN, *2/*2xN) has activity score range (>2.25) (Ryan and Isbister, 2015).

### Data collection

Data were collected from the Leumit patients’ medical records, which incorporate data from multiple sources: primary care physicians, community specialty clinics, hospitalizations, laboratories, and pharmacies. We obtained demographic information, genotyping and phenotyping outcomes, concomitant medications, and the occurrence of CNS and gastrointestinal adverse events known to be associated with tramadol treatment, including dizziness, drowsiness, sedation, headache, seizures, nausea, abdominal pain, decreased appetite, and hyponatremia. We excluded patients with active/chronic gastrointestinal disorders such as acute gastrointestinal infection, celiac, colitis, diverticulosis, gastrointestinal cancers, irritable bowel disease, inflammatory bowel disease, and central nervous system disorders such as acute infection, chronic headache, epilepsy, head injury, or status post stroke, Meniere’s disease, migraine, motion sickness, vestibular neuritis, vestibular disorders, depression and psychiatric disorders were also excluded. In addition, patients prescribed concomitant medications with a proven side effects resembling the same side effects tested during the follow-up period were not included.

### Data analysis

To test the hypothesis that carriers of the CYP2D6*10/*10 are at increased risk of developing nervous system and gastrointestinal adverse effects, we fitted a binary logistic regression model with CYP2D6*10 allele carriage, age, daily tramadol dose, creatinine clearance, and concomitant use of CYP2D6 inhibitors as pre-specified explanatory variables (Vazzana et al., 2015; DeLemos et al., 2017; Al-Qurain et al., 2022). Bootstrapping with 400 repetitions was used for internal validation and to derive bootstrapped 95% confidence intervals. The same method was used to separately estimate the effect of CYP2D6*10 allele carriage on gastrointestinal and nervous system adverse effects. A *p*-value <0.05 was considered statistically significant. Analysis was performed using the ‘rms’ package in R (R Core Team, 2021; Harrell, 2021).

## Results

**FIGURE 1:**
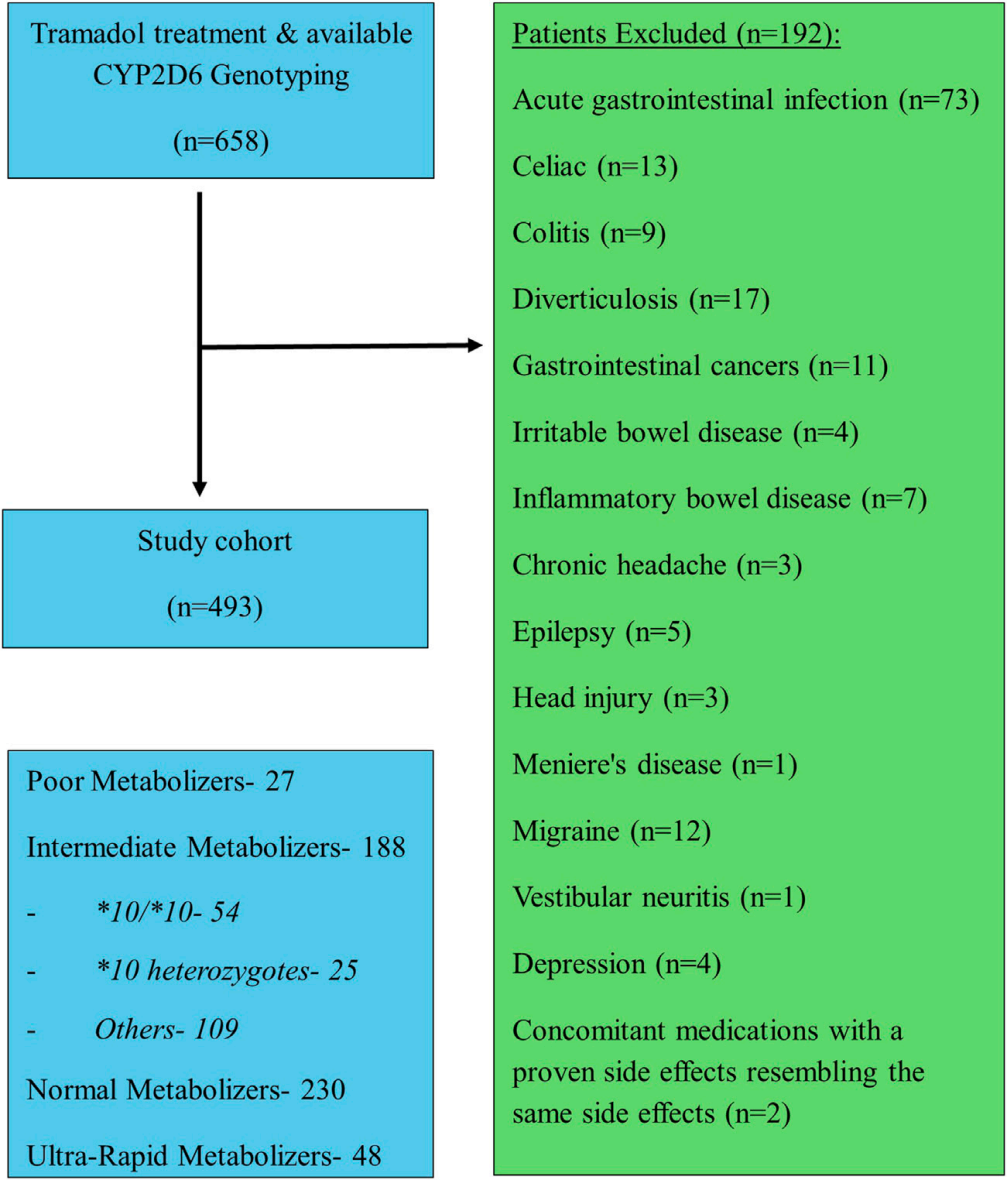
The study diagram.

A total of four hundred ninety-three patients were allocated (Figure 1); among them 56 patients were carriers of the CYP2D6*10/*10 genotype and 437 patients were carriers of other variants of *CYP2D6*. The pain classification of the patients was chronic post-surgical or post-traumatic pain (35%), chronic cancer-related pain that is not related to the central nervous or gastrointestinal system (23%), Chronic neuropathic pain (17%), and Acute pain after injury, surgery, or trauma (25%). The study diagram is attached in Figure 1. The two groups were well-balanced with respect to baseline characteristics (Table 1). The median age was 66 (IQR 58–75) years, and 290 (59%) were women. All patients had a diagnosis of acute or chronic pain leading to tramadol initiation. Most patients (308, 62%) were treated with tramadol immediate release, while 185 (38%) were treated with prolonged release medication. The median duration of tramadol treatment was 30 days (IQR 30–137). Cigarettes smoking was reported in 92 (19%) patients, eight (1.6%) patients used concomitant drugs that may alter CYP2D6 plasma concentration, but non reported use of concomitant herbal products that may inhibit or induce CYP2D6. The median calculated creatinine clearance was 86 mL/min (IQR 64–109). The distribution of *CYP2D6* genotypes is presented in Figure 1. Overall, 56 (11%) patients were homozygous for CYP2D6*10 variant, and only 25 (5.1%) were heterozygous for CYP2D6*10. We focused mainly on patients with the CYP2D6*10/*10 genotype, because of their high prevalence among different populations (Gaedigk et al., 2017) and being ill addressed in previous studies. Of note, 83% of the study participants had a higher CYP2D6 activity score than CYP2D6*10/*10, with only 5% labeled as poor/lower metabolizers.

**TABLE 1:** Basic patient characteristics.

Characteristic	Total (N = 493)	CYP2D6*10/*10 (N = 56)	Other *CYP2D6* genotypes¶ (N = 437)	*p*-value
Female (%)	290 (59)	32 (57)	258 (59)	0.8
Median weight, kilograms (IQR)	76 (67–85)	75 (68–85)	76 (67–85)	0.6
Median age, years (IQR)	66 (58–75)	64 (56–74)	67 (59–76)	0.3
Median height, centimeters (IQR)	163 (155–170)	164 (156–170)	162 (155–170)	0.1
Race or ethnic group (%)				0.4
Jewish	391 (79)	42 (75)	349 (80)	
Arabs	102 (21)	14 (25)	88 (20)	
Smoking (%)				0.9
Yes	92 (19)	11 (20)	81 (19)	
No	367 (74)	45 (80)	322 (74)	
Unknown	34 (6.9)	0 (0)	34 (7.8)	
Residence (%)				1
Urban	459 (93)	52 (93)	407 (93)	
Rural	34 (6.9)	4 (7.1)	30 (6.9)	
Tramadol formulation (%)				0.5
Immediate release	308 (62)	37 (66)	271 (62)	
Prolong release	185 (38)	19 (34)	166 (38)	
Median daily tramadol dose, milligrams (IQR)	100 (100–150)	100 (100–200)	100 (100–150)	0.055
Median duration of therapy, days (IQR)	30 (30–137)	30 (30–175)	30 (30–120)	0.1
Median creatinine clearance, mL/min (IQR)^ϯ^	86 (64–109)	81 (58–107)	87 (65–109)	0.3
Median AST, U/l (IQR)	16 (12–20)	15 (13–20)	16 (12–20)	0.4
Median ALT, U/l (IQR)	14 (10–20)	13 (11–18)	15 (10–21)	1

Abbreviation: IQR, interquartile range.

None of the characteristics differed significantly between the groups (*p* > 0.05 for al comparisons).

¶The distribution of other *CYP2D6* genotypes is presented in Table 4.

^ϯ^ The creatinine clearance was calculated by Cockcroft-Gault formula.

We next evaluated the occurrence of tramadol related adverse effects. Table 2 shows the frequencies of adverse effects in the gastrointestinal and central nervous systems. A total of 403 adverse effects were documented during the study period. Of these, 250 (62%) were reported in the central nervous system and 153 (38%) in the gastrointestinal system. Dizziness, sedated state, headache, and abdominal pain were the most frequent adverse effects, reported in 70 (17.3%), 64 (15.8%), 64 (15.8), and 90 (22.3%) of the patients, respectively. Patients with the CYP2D6*10/*10 genotype had 98 adverse effects, 60 (61.2%) in the central nervous system and 38 (38.8%) in the gastrointestinal system.

**TABLE 2:** Adverse effects in the central nervous and gastrointestinal systems classified by CYP2D6 genotypes.

Adverse effects	CYP2D6 genotype	
	*10/*10 (n = 56)	Other (n = 437)
Central nervous system (%)	60	190
Dizziness	21 (38%)	49 (11%)
Drowsiness	11 (20%)	32 (7%)
Sedated state	21 (38%)	43 (10%)
Headache	5 (9%)	59 (14%)
Seizure	2 (4%)	7 (2%)
Gastrointestinal system (%)	38	115
Nausea	14 (25%)	38 (9%)
Abdominal pain	19 (34%)	71 (16%)
Decreased appetite	5 (9%)	6 (1%)

Compared to other genotypes, and after adjustment for other variables possibly related to increased adverse effects, including age, daily tramadol dose, creatinine clearance, and concomitant use of CYP2D6 inhibitors, patients with CYP2D6*10/*10 genotype were 6.14 times more likely to report an adverse event (95% confidence interval [CI], 3.18–11.83). They had also higher odds for adverse events than patients tested heterozygous to CYP2D6*10 and other intermediate metabolizers group, OR 5.73 (95% CI, 2.41–14.36), *p* < 0.001, and OR 0.13 (95% CI, 0.06–0.28), *p* < 0.001, respectively. The odds of adverse events in the central nervous system and the gastrointestinal system were 5.13 times (95% CI, 2.84–9.28) and 3.25 times (95% CI, 1.78–5.93) higher in patients with the CYP2D6*10/*10 genotype (Table 3). Concomitant drugs with tramadol that inhibit or induce CYP2D6 did not affect the study results (OR = 0.63, 95% CI, 0.19–2.13). Notably, pain levels were not routinely tracked through the treatments course, and the retrospective nature of the study precludes pain relief quantification; nevertheless, Table 1 shows that the median daily tramadol dose, in milligrams, was comparable between CYP2D6*10/*10 genotype and the others.

**TABLE 3:** Multivariable logistic regression: The effect of CYP2D6*10/*10 genotype on the odds for adverse effects.

Outcomes	CYP2D6*10/*10 genotype (N = 56) n (%)	Other *CYP2D6* genotypes (N = 437) n (%)	Adjusted odds ratio^a^ (95% CI)	*p*-value^2^
Primary outcome				
Adverse effects in central nervous or gastrointestinal systems	43 (76.8)	153 (35.0)	6.14 (3.18–11.83)	*p* < 0.001
Secondary outcomes				
Adverse effects in central nervous system	35 (62.5)	104 (23.8)	5.13 (2.84–9.28)	*p* < 0.001
Adverse effects in gastrointestinal system	23 (41.1)	79 (18.1)	3.25 (1.78–5.93)	*p* < 0.001

Abbreviation: CI, confidence interval.

^a^ Adjusted for age, daily tramadol dose, creatinine clearance and concomitant use of CYP, 2D6 inhibitors.

2*p*<0.05 was considered statistically significant.

**TABLE 4:** CYP2D6 genotypes distribution, n (%).

*10/*10	56 (11)
*1/*1	55 (11)
*1/*2A	66 (13)
*1/*4	45 (9.1)
*1/*41	51 (10.3)
*10/*41	7 (1.4)
*2A/*10	10 (2.0)
*2A/*2A	15 (3.0)
*2A/*4	10 (2.0)
*2A/*41	10 (2.0)
*4/*4	14 (2.8)
*4/*41	19 (3.9)
*1/*10	2 (0.4)
*1/*17	9 (1.8)
*1/*3	3 (0.6)
*1/*35	9 (1.8)
*1/*5	3 (0.6)
*1/*6	5 (1.0)
*1/*9	1 (0.2)
*10/*17	3 (0.6)
*17/*41	1 (0.2)
*2/*41	8 (1.6)
*2A/*17	1 (0.2)
*2A/*3	3 (0.6)
*2A/*35	3 (0.6)
*2A/*5	1 (0.2)
*2A/*9	1 (0.2)
*3/*35	1 (0.2)
*3/*4	9 (1.8)
*3/*41	2 (0.4)
*4/*10	1 (0.2)
*4/*17	1 (0.2)
*4/*35	6 (1.2)
*4/*5	2 (0.4)
*4/*6	1 (0.2)
*4/*7	1 (0.2)
*4/*9	1 (0.2)
*41/*41	6 (1.2)
*7/*41	2 (0.4)
*9/*10	1 (0.2)
*1/*1 with duplication	1 (0.2)
*1/*2A with duplication	12 (2.4)
*2/*10 with duplication	1 (0.2)
*2/*35 with duplication	11 (2.2)
*2A/*2A with duplication	17 (3.4)
*2A/*35 with duplication	3 (0.6)
*2A/*41 with duplication	3 (0.6)

## Discussion

Although tramadol is widely used for pain alleviation, information on its pharmacokinetics is scarce. Tramadol is available as a racemic mixture with different mechanistic effects of its (+)/(−) enantiomers, and its primary CYP2D6 derived metabolite, *O*-desmethyltramadol (ODT). The latter has a 200-fold greater affinity for µ-opioid receptors as compared with the parent tramadol (Al-Qurain et al., 2022). The (+) enantiomer has a weak affinity for the μ-receptor and effectively inhibits serotonin reuptake, whereas the (−) enantiomer is a more effective inhibitor of noradrenaline reuptake. Thus, the antinociceptive effect of tramadol is achieved through central opioid receptor–mediated effect (by ODT and (+) enantiomer), and serotonin and norepinephrine reuptake inhibition (by the (+) and (−) enantiomers, respectively). Since the primary pharmacologically active metabolite ODT is formed by CYP2D6, genetic polymorphism of the CYP2D6 were repeatedly linked to tramadol response, however, with a conflicting data.

CYP2D6 low metabolizers have been shown to have lower median plasma levels of the active metabolite) ODT) after a dose of tramadol as compared with higher metabolizers, and they are more often resistant to tramadol`s analgesic effect (R Core Team, 2021; Harrell, 2021; Ardakani et al., 2008; Wang et al., 2006; Stamer et al., 2003; Swen et al., 2011; Dong et al., 2015). On the other hand, rapid metabolizers display higher peak plasma concentrations of ODT after tramadol administration, accompanied by greater analgesia, and higher incidence of adverse events as compared with lower metabolizers (Wang et al., 2006; Wen et al., 2020). The above observation might reflect a reduced efficacy of tramadol in low CYP2D6 metabolizers, and reduced tolerability in rapid metabolizers. In contrary, CYP2D6*10/*10 metabolizers were elsewhere linked to higher levels of tramadol and its active metabolite (ODT) compared to the wild-type phenotype (Lee et al., 2019), and higher sedative effect and efficacy (Ijichi et al., 2005; Seripa et al., 2015). A common feature of all the previous studies is a short-lasting follow up and/or small number of patients included.

Intriguingly, our real-life data provide evidence for higher incidence of tramadol-related adverse events in patients tested positive for the CYP2D6*10/*10 variant. To the best of our knowledge, this is the first study to assess the effect of the CYP2D6*10/*10 genotype compared to other genotypes regarding side effects among long-term tramadol users in a longitudinal real-life study. We show that patients with the CYP2D6*10/*10 variant sustain higher incidence of side events as compared with other CYP2D6 genotypes despite similar median tramadol day dose. Overall, we believe *10/*10 subjects exert their clinical effect primarily by a non-central opioid effect. Nonetheless, the comparable adverse effects profile between *10/*10 subjects and the rest of intermediate metabolizer proves that intermediate metabolizers as a rule exhibit a higher adverse events profile than most metabolizers. The high prevalence of *10/*10 genotype among different populations makes it an important potential screening target. Our results were neither affected by the renal function nor by any CYP2D6 induction agent. Despite former studies documenting higher tramadol consumption among low metabolizers, our long-term follow up documented similar tramadol consumption among CYP2D6*10/*10 carriers. We neither scored pain response nor tested the usage of other classes of anti-pain medications; we also did not test plasma tramadol and/or ODT levels, however, we are aware that tramadol has dual analgesic mechanisms: activation of opioid receptors and enhancement of serotonin and norepinephrine transmission. A previous population pharmacokinetic study of tramadol and its metabolites used in healthy Korean subjects showed that the peak plasma concentration of tramadol for CYP2D6*10/*10 carriers, was approximately 1.5 times higher than that of the wild type at the steady state after multiple tramadol 100 mg twice daily administration (Lee et al., 2019). Additionally, the clearance of the parent drug is estimated as 16.9 L/h for the wild type and 11 L/h for the CYP2D6*10/*10 group, while the clearance of the active metabolite (ODT) is estimated as 4.11 L/h for wild type and 1.94 L/h for CYP2D6*10/*10 group. The higher levels of ODT, and the (+) and (−) tramadol enantiomers, in conjunction with slower clearance of ODT and tramadol most probably contributed to the analgesic effect among CYP2D6*10/*10 carriers. The higher occurrence of AEs is a result of long-term tramadol treatment, and probably the higher levels of the enantiomers and the major metabolite with a slower clearance from the circulation (Lee et al., 2019).

Future clinical studies with pharmacokinetic measurements should follow our results, including testing the association between the CYP2D6 genotypes, drug level in the blood and both the treatment efficacy and tolerability. In addition, we must not underscore a possible contribution of genetic variability in the organic cation transporter OCT1 to the pharmacokinetics of tramadol (Tzvetkov et al., 2017). Polymorphism in OCT1 can potentially affect tramadol’s efficacy. This factor could be considered for additional analysis.

### Study limitations

Several limitations of the study worth mentioning; first, the relatively small sample size of patients. Second, the lack of data regarding the plasma concentrations of both tramadol and its active metabolite (ODT). Additionally, despite a binary logistic regression model that accounted for relevant potential confounders, some residual confounding might exist due to the study’s retrospective design. The severity of the pain and environmental components that may affect the likelihood of experiencing adverse effects are out of the scope of our retrospective study. Finally, a causal relation between CYP2D6*10/*10 genotype and the occurrence of adverse effects cannot be generated due to the observational design of the study.

## Conclusion

The results of this study suggest that patients with the CYP2D6*10/*10 genotype treated with tramadol have a significantly increased risk of experiencing adverse effects. The results support the potential clinical utility of CYP2D6 genotyping in populations where there is a high prevalence of the CYP2D6*10/*10 genotype before starting tramadol treatment or prescribing an alternative drug that is less dependent on CYP2D6 to minimize the risk of adverse effects and therapeutic failure. Future clinical studies with pharmacokinetic measurements should follow our results, including testing the association between the CYP2D6 genotypes and the drug level in the blood and both the drug efficacy and tolerability.

## Impact statements of findings on practice

• The extensive heterogeneity in CYP2D6 expression largely affect the metabolism of tramadol.
• Surprisingly, the decreased-function CYP2D6*10/*10 genotype is associated with higher occurrence of tramadol related adverse events, primarily related to the central nervous and gastrointestinal systems.
• Personalized tramadol prescription based on genotype screening is advocated to mitigate adverse events occurrence and enhance safety.

## Data Availability

The data that support the findings of this study are stored in Leumit Health Services (LHS) database, but restrictions apply to the availability of these data, which were used under license for the current study, and so are not publicly available. Access to raw patient data is restricted to researchers approved by the institutional ethics committee. Data are however available from the authors upon reasonable request and upon the regulation of the Israeli Ministry of Health and approval of the research institute of LHS. Requests to access these datasets should be directed to the corresponding author, RA Fanne at rabufanne@gmail.com.

## References

[B1] Al-Qurain A. A. Upton R. N. Tadros R. Roberts M. S. Wiese M. D. (2022). Population pharmacokinetic model for tramadol and O-desmethyltramadol in older patients. Eur. J. Drug Metab. Pharmacokinet. 47 (3), 387–402. Epub 2022 Feb 15. PMID: 35167052; PMCID: PMC9050769. 10.1007/s13318-022-00756-x 35167052 PMC9050769

[B2] Ardakani Y. H. Mehvar R. Foroumadi A. Rouini M. R. (2008). Enantioselective determination of tramadol and its main phase I metabolites in human plasma by high-performance liquid chromatography. J. Chromatogr. 864, 109–115. 10.1016/j.jchromb.2008.01.038 18282749

[B3] Azhar Gohar W. J. Ashcraft K. Neradilek M. B. Newman R. L. Thirumaran R. K. Moyer N. (2016). Differences in medicare quality measures among nursing homes after pharmacogenetic testing. J. Res. Dev. 4 (1).

[B4] Bradford L. D. (2002). CYP2D6 allele frequency in European Caucasians, Asians, Africans and their descendants. Pharmacogenomics 3 (2), 229–243. 10.1517/14622416.3.2.229 11972444

[B5] Caudle K. E. Sangkuhl K. Whirl-Carrillo M. Swen J. J. Haidar C. E. Klein T. E. (2020). Standardizing CYP2D6 genotype to phenotype translation: consensus recommendations from the clinical pharmacogenetics implementation consortium and Dutch pharmacogenetics working group. Clin. Transl. Sci. 13 (1), 116–124. Epub 2019 Oct 24. 10.1111/cts.12692 31647186 PMC6951851

[B6] Crews K. R. Monte A. A. Huddart R. Caudle K. E. Kharasch E. D. Gaedigk A. (2021). Clinical pharmacogenetics implementation consortium guideline for CYP2D6, OPRM1, and COMT genotypes and select opioid therapy. Clin. Pharmacol. Ther. 110 (4), 888–896. Epub 2021 Feb 9. PMID: 33387367; PMCID: PMC8249478. 10.1002/cpt.2149 33387367 PMC8249478

[B7] DeLemos B. Richards H. M. Vandenbossche J. Ariyawansa J. Natarajan J. Alexander B. (2017). Safety, tolerability, and pharmacokinetics of therapeutic and supratherapeutic doses of tramadol hydrochloride in healthy adults: a randomized, double-blind, placebo-controlled multiple-ascending-dose study. Clin. Pharmacol. Drug Dev. 6 (6), 592–603. Epub 2017 Sep 7. PMID: 28881493. 10.1002/cpdd.378 28881493

[B8] Dong H. Lu S. J. Zhang R. Liu D. D. Zhang Y. Z. Song C. Y. (2015). Effect of the CYP2D6 gene polymorphism on postoperative analgesia of tramadol in Han nationality nephrectomy patients. Eur. J. Clin. Pharmacol. 71, 681–686. 10.1007/s00228-015-1857-4 25948472

[B9] Faria J. Barbosa J. Moreira R. Queirós O. Carvalho F. Dinis‐Oliveira R. (2018). Comparative pharmacology and toxicology of tramadol and tapentadol. Eur. J. Pain 22 (5), 827–844. 10.1002/ejp.1196 29369473

[B10] Gaedigk A. Sangkuhl K. Whirl-Carrillo M. Klein T. Leeder J. S. (2017). Prediction of CYP2D6 phenotype from genotype across world populations. Genet. Med. 19 (1), 69–76. 10.1038/gim.2016.80 27388693 PMC5292679

[B11] Harrell F. E. Jr (2021). RMS: regression modeling strategies. R package version 6.2-0. Available at: https://CRAN.R-project.org/package=rms (Accessed September 25, 2023).

[B12] Ijichi K. Nijima K. Iwagaki T. Irie J. Uratsuji Y. (2005). A randomized double-blind comparison of epidural versus intravenous tramadol infusion for postoperative analgesia. Masui. Jpn. J. Anesthesiol. 54, 615–621. 15966377

[B13] Kirchheiner J. Keulen J. T. Bauer S. Roots I. Brockmöller J. (2008). Effects of the CYP2D6 gene duplication on the pharmacokinetics and pharmacodynamics of tramadol. J. Clin. Psychopharmacol. 28, 78–83. 10.1097/JCP.0b013e318160f827 18204346

[B15] Koopmans A. B. Braakman M. H. Vinkers D. J. Hoek H. W. van Harten P. N. (2021). Meta-analysis of probability estimates of worldwide variation of CYP2D6 and CYP2C19. Transl. Psychiatry 11 (1), 141. 10.1038/s41398-020-01129-1 33627619 PMC7904867

[B16] Lee J. Yoo H. D. Bae J. W. Lee S. Shin K. H. (2019). Population pharmacokinetic analysis of tramadol and O-desmethyltramadol with genetic polymorphism of CYP2D6. Drug Des. Devel Ther. 13, 1751–1761. 10.2147/DDDT.S199574 PMC6537040 31213765

[B17] López-Flores L. A. Pérez-Rubio G. Falfán-Valencia R. (2017). Distribution of polymorphic variants of CYP2A6 and their involvement in nicotine addiction. EXCLI J. 16, 174–196. 10.17179/excli2016-847 28507465 PMC5427481

[B18] Luo H. R. Aloumanis V. Lin K. M. Gurwitz D. Wan Y. J. Y. (2004). Polymorphisms of CYP2C19 and CYP2D6 in Israeli ethnic groups. Am. J. Pharmacogenomics 4, 395–401. 10.2165/00129785-200404060-00006 15651900

[B19] Mohammadpour A. Ashkezari M. D. Farahmand B. Shokrzadeh M. (2019). Demographic characteristics and functional performance of the kidneys and hearts of patients with acute tramadol toxicity. Drug Res. 69, 207–210. 10.1055/a-0646-3918 29996175

[B20] R Core Team (2021). R: a language and environment for statistical computing. Vienna, Austria: R Foundation for Statistical Computing.

[B21] Ryan N. M. Isbister G. K. (2015). Tramadol overdose causes seizures and respiratory depression but serotonin toxicity appears unlikely. Clin. Toxicol. 53 (6), 545–550. 10.3109/15563650.2015.1036279 25901965

[B22] Saarikoski T. Saari T. I. Hagelberg N. M. Backman J. T. Neuvonen P. J. Scheinin M. (2015). Effects of terbinafine and itraconazole on the pharmacokinetics of orally administered tramadol. Eur. J. Clin. Pharmacol. 71 (3), 321–327. 10.1007/s00228-014-1799-2 25560051

[B23] Seripa D. Latina P. Fontana A. Gravina C. Lattanzi M. Savino M. (2015). Role of CYP2D6 polymorphisms in the outcome of postoperative pain treatment. Pain Med. Malden, Mass 16, 2012–2023. 10.1111/pme.12778 25989235

[B24] Stamer U. M. Lehnen K. Höthker F. Bayerer B. Wolf S. Hoeft A. (2003). Impact of CYP2D6 genotype on postoperative tramadol analgesia. Pain 105, 231–238. 10.1016/s0304-3959(03)00212-4 14499440

[B25] Stamer U. M. Musshoff F. Kobilay M. Madea B. Hoeft A. Stuber F. (2007). Concentrations of tramadol and O-desmethyltramadol enantiomers in different CYP2D6 genotypes. Clin. Pharmacol. Ther. 82, 41–47. 10.1038/sj.clpt.6100152 17361124

[B26] Stassinos G. L. Gonzales L. Klein-Schwartz W. (2017). Characterizing the toxicity and dose-effect profile of tramadol ingestions in children. Pediatr. Emerg. Care 35, 117–120. 10.1097/PEC.0000000000001084 28225374

[B27] Swen J. J. Nijenhuis M. de Boer A. Grandia L. Maitland-van der Zee A. H. Mulder H. (2011). Pharmacogenetics: from bench to byte--an update of guidelines. Clin. Pharmacol. Ther. 89, 662–673. 10.1038/clpt.2011.34 21412232

[B28] Tzvetkov M. V. (2017). OCT1 pharmacogenetics in pain management: is a clinical application within reach? Pharmacogenomics 18, 1515–1523. 10.2217/pgs-2017-0095 29061087

[B29] Vazzana M. Andreani T. Fangueiro J. Faggio C. Silva C. Santini A. (2015). Tramadol hydrochloride: pharmacokinetics, pharmacodynamics, adverse side effects, co-administration of drugs and new drug delivery systems. Biomed. Pharmacother. 70, 234–238. Epub 2015 Feb 7. PMID: 25776506. 10.1016/j.biopha.2015.01.022 25776506

[B30] Wang G. Zhang H. He F. Fang X. (2006). Effect of the CYP2D6*10 C188T polymorphism on postoperative tramadol analgesia in a Chinese population. Eur. J. Clin. Pharmacol. 62, 927–931. 10.1007/s00228-006-0191-2 16960721

[B31] Wen Q. H. Zhang Z. Cai W. K. Lin X. Q. He G. H. (2020). The associations between CYP2D6*10 C188T polymorphism and pharmacokinetics and clinical outcomes of tramadol: a systematic review and meta-analysis. Pain Med. (Malden, Mass) 21, 3679–3690. 10.1093/pm/pnaa140 32488232

[B32] Wu W. McKown L. Liao S. (2002). Metabolism of the analgesic drug ULTRAM (tramadol hydrochloride) in humans: API-MS and MS/MS characterization of metabolites. Xenobiotica 32 (5), 411–425. 10.1080/00498250110113230 12065063

